# Compound Kushen Injection Protects Skin From Radiation Injury *via* Regulating Bim

**DOI:** 10.3389/fphar.2021.753068

**Published:** 2021-12-09

**Authors:** Jianxiao Zheng, Gong Li, Juanjuan Wang, Shujing Wang, Qing Tang, Honghao Sheng, Wanyin Wu, Sumei Wang

**Affiliations:** ^1^ Department of Oncology, Clinical and Basic Research Team of TCM Prevention and Treatment of NSCLC, the Second Clinical College of Guangzhou University of Chinese Medicine, Guangdong Provincial Hospital of Chinese Medicine, Guangdong Provincial Key Laboratory of Clinical Research on Traditional Chinese Medicine Syndrome, Guangdong-Hong Kong-Macau Joint Lab on Chinese Medicine and Immune Disease Research, Guangzhou University of Chinese Medicine, Guangzhou, China; ^2^ Department of Radiology, Guangdong Provincial Hospital of Chinese Medicine, the Second Clinical Medical College of Guangzhou University of Chinese Medicine, Guangzhou, China; ^3^ State Key Laboratory of Dampness Syndrome of Chinese Medicine, the Second Affiliated Hospital of Guangzhou University of Chinese Medicine, Guangzhou, China; ^4^ The Second Clinical College of Guangzhou University of Chinese Medicine, Guangzhou, China

**Keywords:** compound kushen injection, radiation injury, skin, cancer, Bim

## Abstract

**Background:** Radiation-induced skin injury is a major side-effect observed in cancer patients who received radiotherapy. Thus identifying new radioprotective drugs for prevention or treatment of post-irradiation skin injury should be prompted. A large number of clinical studies have confirmed that Compound Kushen injection (CKI) can enhance efficacy and reduce toxicity of radiotherapy. The aim of this study is to confirm the effect of CKI in alleviating radiotherapy injury in the skin and explore the exact mechanism.

**Methods:** 60 patients who met the inclusion/exclusion criteria were allocated to treatment group (CKI before radiotherapy) or control group (normal saline before radiotherapy) randomly. MTT assay, flow cytometry, Western Blot, and transient transfection were performed to detect the cell viability, cell apoptosis and Bim expression after treatment with CKI or/and radiotherapy.

**Results:** CKI had the effect of alleviating skin injury in cancer patients who received radiotherapy in clinic. CKI induced cancer cell apoptosis when combined with irradiation (IR), while it reversed the induction of cell apoptosis by IR in human skin fibroblast (HSF) cells. And Bim, as a tumor suppressor, was induced in cancer cells but had no change in HSF cells when treated with CKI. Moreover, the above effect could be attenuated when Bim was silenced by siRNA.

**Conclusion:** We conclude that CKI represents a promising radio-protective agent with a potential differential beneficial effect on both cancer cells (inducing apoptosis) and HSF cells (providing radio-protection *via* inhibiting IR-induced apoptosis), via regulating Bim. Our study uncovers a novel mechanism by which CKI inhibits human cancer cell while protects skin from radiotherapy, indicating CKI might be a promising radio-protective drug.

**Clinical Trial Registration:** Chinese Clinical Trial Registry (www.chictr.org.cn), identifier ChiCTR2100049164.

## Introduction

Radiation therapy is an effective non-surgical treatment for human cancers. However, the radiation injury induced by radiation therapy affects the quality of life (QoL) of patients inevitably and seriously. Therefore, prevention and treatment of radiation injury is of great significance. Compound Kushen injection (CKI) is the most used Chinese Herbal injections (CHIs) in human cancers. It has been reported to have the ability to increase QoL and enhance immunity of cancer patients, especially when patients received radiotherapy (Yang et al., 2021). For example, a study showed that CKI combined with radiotherapy is the most preferable and beneficial option for patients with esophageal cancer (EC) in terms of efficacy and safety (Zhang et al., 2019). Since CKI could improve the clinical effectiveness rate and performance status of cancer patients, whether CKI could relieve the radiation injury in the skin at the same time is to be illustrated.

CKI, a Traditional Chinese Medicine (TCM) preparation and a mixture of natural compounds extracted from Kushen (*Sophorae Flavescentis Radix*) and Tufuling (*Smilacis Glabrae Rhixoma*), which have been used for adjuvant anti-cancer clinical therapy for over 20 years (Qu et al., 2016; Cui et al., 2020). Based on the TCM theory, CKI can be used for clearing away heat and dampness, cooling blood and detoxifying and relieving cancer pain, which has been proven to have significant effects against cancer (Guo et al., 2015; Yu et al., 2017). Based on the Western Medicine (WM) theory, CKI suppressed tumor growth by inhibiting cancer cell proliferation and metastasis, promoting cell apoptosis and improving patients’ immune system (Wang H. et al., 2019). For example, CKI-primed macrophages obviously promoted the proliferation and the cytotoxic ability of CD8^+^ T cells and decreased its exhaustion, resulting in HCC (hepatocellular carcinoma) cell apoptosis (Yang et al., 2020). Another study reported that CKI inhibited HCC progression by regulating signaling pathways involving MMP2 (Matrix Metalloproteinase-2) and Caspase-3 and the key pathways of glycometabolism and amino acid metabolism (Gao et al., 2018).

There were researches revealed that the active ingredients from CKI could induce cancer cell apoptosis by regulating p53 and PI3K-Akt pathway (He et al., 2020). Matrine and oxymatrine are main components from *Sophorae Flavescentis Radix*. It has been reported that both matrine and oxymatrine have characteristics of anti-inflammatory, anti-tumor, anti-viral, and cardiovascular protection effects (Ma et al., 2014; Wang et al., 2015). Matrine could suppress the BrCSCs (breast cancer stem cells) differentiation and self-renewal by downregulating Lin28A expression, leading to the inactivation of Wnt pathway in a Let-7b-dependent way (Li et al., 2020). Oxymatrine could inhibit the invasiveness of HCC by reducing the expression of MMP-2/-9 *via* inhibiting the activity of p38 signaling pathway (Chen et al., 2019). Meanwhile, *Smilacis Glabrae Rhixoma* has also been reported to have anti-inflammatory, antiviral, anti-cancer, and immunomodulatory effects (Jiang and Xu, 2003; Ooi et al., 2004; Galhena et al., 2012; Samarakoon et al., 2012). For instance, it could suppress the phosphorylation of Akt (Thr308) and thereby inhibit gastric cancer (GC) cell proliferation and metastasis as well as accelerating GC cell apoptosis through Akt-mediated signaling pathways (Hao et al., 2016).

The above studies emphasized the inhibition role of CKI in human cancers. What’s the role of CKI in non-cancerous cells? A previous study showed that cell apoptosis was increased by CKI in breast cancer (BC) but not in non-cancerous lines (Nourmohammadi et al., 2019). Interestingly, our clinic data also showed that CKI had the effect of protecting skin from radiation injury in patients with nasopharyngeal carcinoma (NPC) who received radiotherapy. Therefore, we hypothesized that CKI has differential roles in human cancer and skin cells. In the present study, we’ll further identify the role and mechanism of CKI in human cancers including lung cancer (LC) and NPC when combined with radiotherapy, and in human skin fibroblast (HSF) cells.

## Materials and Methods

### Clinical Research Protocol

The clinical research protocol was approved by the Ethics Committee of Guangdong Provincial Hospital of Traditional Chinese Medicine (YF2018-064). Sixty-two patients from the department of Radiotherapy, Guangdong Provincial Hospital of Traditional Chinese Medicine, were identified, screened, and enrolled in the study between June 2014 and January 2018 and all patients provided informed consent. The factors affecting the severity of acute radiation dermatitis, such as age, obesity, KPS (Karnofsky Performance Status) score, stage and other baseline characteristics, were comparable between treatment group and control group. If the subjects would like to withdrawal of consent or fail to adhere to the research protocol or serious adverse events happened, the study of the participant would be suspended and recorded as withdrawn. During the treatment, two patients withdrew from the clinical trial due to personal wishes, and the other sixty patients successfully completed the trial as planned.

### Inclusion/Exclusion Criteria

#### Inclusion Criteria

• Patients who were pathologically diagnosed as Nasopharyngeal Carcinoma (NPC) and planned to receive intensity modulated radiotherapy (IMRT).
• The age is between 18 and 70 years old, regardless of gender.
• Normal bone marrow, liver, kidney, heart, and lung function.
• Patients received the first radical radiotherapy or chemo-radiotherapy.
• Karnofsky’s behavior score is above 60.

#### Exclusion Criteria

• Active repeated cancer.
• Patients suffering from mental disorders.
• Patients with skin infectious diseases.
• Allergic constitution or allergic to the components of the preparation.
• Suspected or true history of alcohol and drug abuse.
• Pregnant or lactating women or those with recent birth planning.
• The researcher believes that it is not suitable to participate in this experiment.

### Study Design

In the prospective trial, 60 patients who met the inclusion/exclusion criteria were allocated to treatment group or control group using a computer-generated random sequence.

### The Radiation-Induced Skin-Reaction-Assessment-Scale Score

The skin of the neck radiation field was evaluated weekly from baseline using the RISRAS table for a total of 7 weeks. The first part of the RISRAS form is filled in by the patient, which is the subjective symptom evaluation form of the patient, including itching, pain, burning or tension of the skin of the neck radiation field, and the impact of the skin reaction caused by radiotherapy on the patient’s daily activities. The patient’s response ranges from “none at all” to “very.” The higher the score, the more serious the patient’s subjective symptoms (Supplementary Table S1). The second part is the professional scoring table for medical staff, including erythema (E), dry desquamation (DD), wet desquamation (WD), and necrosis (N). “E” is scored according to the change of skin color of the patient’s neck irradiation field, and 0–4 points from “normal” to “deep purple erythema.” For “DD,” “WD,” and “N” the proportion of the area of dermatitis in the whole radiation field skin was evaluated, and 0–4 points were calculated from “normal” to “>75%–100” (Supplementary Table S2). Finally, the test observer will summarize the scores of the two scoring tables into the continuous evaluation table to get a final score of each patient.

### Intervention

All candidates were treated with standard intensity modulated radiotherapy according to the NPC guidelines of Chinese society of clinical oncology (CSCO). In the treatment group, the candidates received 10 ml of CKI solution intravenously each day before radiotherapy. In the control group, the candidates received normal saline solution intravenously each day before radiotherapy.

#### Trial Registration

Chinese Clinical Trial Registry Trial registration number: ChiCTR2100049164. Name of registry: Compound Kushen injection alleviates radiation-induced skin injury: randomized controlled trial. Date of registration: 2021-07-24 (1008002 retrospective registration).

### Chemicals and Cell Culture

Compound Kushen Injection (CKI, z14021231) was produced by Shanxi Zhendong Pharmaceutical Co., Ltd. (Shanxi, China), and 1 ml CKI contains 0.4 g crude drug. MTT (M5655) and Dimethyl sulfoxide (DMSO) were purchased from Sigma-Aldrich Co. (St. Louis, MO, United States). Monoclonal antibodies specific of Bim, and GAPDH were purchased from Cell Signaling Technology Inc. (Beverly, MA, United States). Lipofectamine 3000 reagent was purchased from Life Technologies (Carlsbad, CA, United States). NSCLC (non-small cell lung caner) cell line H1299 and Human Skin Fibroblast (HSF) cell line were obtained from the Chinese Academy of Sciences Cell Bank of Type Culture Collection (Shanghai, China). Nasopharyngeal Carcinoma cell line CNE-2 was purchased from the Cancer Center of Sun Yat-Sen University (Guangzhou, China). All cells were grown at 37°C, in a humidified 5% CO_2_ and 95% air and cultured in RPMI-1640 medium (Life Technologies, Carlsbad, CA, United States) containing 10% FBS (Gibco, United States) and 0.5% penicillin-streptomycin sulfate (Invitrogen Life Technologies, Carlsbad, CA, United States). Cells were counted using the automated cell counter star (Inno-Alliacne Biotech Inc., Denver, CO, United States).

### MTT Assay

MTT assay was performed as described previously (Wang S. et al., 2019). Briefly, cells were seeded in 96-well plates at a density of 1 × 10^4^ cells/well and incubated at 37°C for 24 h. After treatment, cells were maintained at 37°C in a humidified atmosphere containing 5% CO_2_ for 24 h. The medium was supplemented with 20 µL MTT (5 mg/ml) at 37°C for 4 h. Subsequently, the medium was replaced with 100 µL DMSO. Following incubation for 20 min at room temperature, the absorbance was read by measuring the optical density (OD) at 490 nm in a microplate reader (Molecular Devices, LLC, Sunnyvale, CA, United States). The viability rate was calculated as follows: Viability rate (%) = OD 490 trail/OD 490 blank × 100. The experiment was repeated three times.

### Ionizing Radiation

Irradiation was performed at The Second Clinical College of Guangzhou University of Chinese Medicine/Guangdong Provincial Hospital of Traditional Chinese Medicine using MultiRad 225 machine (Faxitron). The cells were divided into the following three groups: Control group, which was cultured in regular medium and without any treatment；Irradiation group, which was treated with irradiation alone; and CKI combined with irradiation group, which was incubated with CKI before irradiation. After irradiation, cells were maintained at 37°C in a humidified atmosphere containing 5% CO_2_ for 24 h for further analysis.

### TUNEL Assay

TUNE assay was performed using TUNEL cell apoptosis kit according to the manufacturer’s protocol (Beyotime, China). Briefly, cells were fixed with paraformaldehyde for 30 min, and incubated at room temperature with 0.3% Trinton X-100 PBS for 5 min. Wash cells with PBS twice. Samples were added with 50 μL TUNEL detect liquid and incubated at 37°C for 60 min in dark. Finally, samples were sealed with antifluorescence quenched liquid and observed under a fluorescence microscope (Nikon ECLIPSE Ti2-E, Japan).

### Confocal Assay of Cell Apoptosis

Cell apoptosis was carried out using cell apoptosis kit according to the manufacturer’s protocol (Dojindo, Japan). Briefly, cells were collected, centrifuged for 3 min at 1,000 rpm, and resuspended in 1 × Annexin V binding solution to a final concentration of 1 × 10^6^ cells/ml. Finally, 5 μL Annexin V-FITC and 5 μL PI were added into the cells at room temperature for 15 min. Then 400 μL 1 × Annexin V binding solution were added. The cells were then analyzed using confocal microscope in 1 h (Zeiss LSM 710, German).

### Flow Cytometry Analysis

Flow cytometry was performed as described previously (Wang et al., 2018). Cell apoptosis was analyzed by Annexin V-FITC/PI apoptosis detection kit according to the manufacturer’s protocol (Sigma-Aldrich Co. St. Louis, MO). Briefly, cells (CNE-2, H1299, and HSF) were seeded in 6-well plates. After 24 h of culture, cells were treated with CKI, IR, or IR combined with CKI, and then incubated at 37°C for 24 h. Afterwards, cells were collected, centrifuged for 5 min at 1,500 rpm, and resuspended in 1 × binding buffer. Finally, 5 μL Annexin V-FITC and 5 μL PI were added into the cells at room temperature for 15 min. The cells were then analyzed using flow cytometer (Beckman FC 500, Beckman Coulter, Inc., CA, United States).

### Western Blot Analysis

Western blot analysis was performed as described previously (Wang et al., 2020). Briefly, cells (CNE-2, H1299, and HSF) were harvested, washed and lysed with 1 × RAPI buffer. Protein concentration was determined by the Thermo BCA protein assay Kit. Equal amounts of protein from cell lysates were solubilized in 5 × SDS sample buffer and separated on 8–10% SDS polyacrylamide gels, and transferred onto polyvinylidene fluoride membranes. Membranes were blocked with 5% non-fat milk in TBST and incubated with primary antibodies against Bim and GAPDH proteins at 4°C overnight. Afterwards, the membranes were washed and incubated with a secondary antibody against rabbit IgG for 1 h, followed by washing and transferring into ECL solution (Millipore, Darmstadt, Germany), and scanned under the Bio-Rad ChemiDoc XRS + Chemiluminescence imaging system (Bio-Rad, Hercules, CA, United States). The results were measured by ImageJ software.

### Transient Transfection Assays

The cells were seeded in 6-well plates and reached to 50–60% confluence. The negative control and Bim siRNA were obtained from RiboBio (Guangzhou, China). For each well, 10–60 nM NC or Bim siRNA were transfected into the cells using Lipofectamine 3000 reagent (Life Technologies, Carlsbad, CA, United States) for 24 h based on the instruction from the provider.

### Statistical Analysis

Statistical analysis was performed using the SPSS statistical software. Statistical evaluation for data analysis used Student’s t-test when there were only two groups (two sided) and differences between groups were assessed by one-way ANOVA. RIAPAS data was evaluated using Wilcoxon matched-pairs signed rank test. All data are reported as Means ± SD. Differences between groups were considered significant statistically when *p* ≤ 0.05.

## Results

### CKI Alleviated Radiotherapy Injury in the Skin in Clinic

**FIGURE 1:**
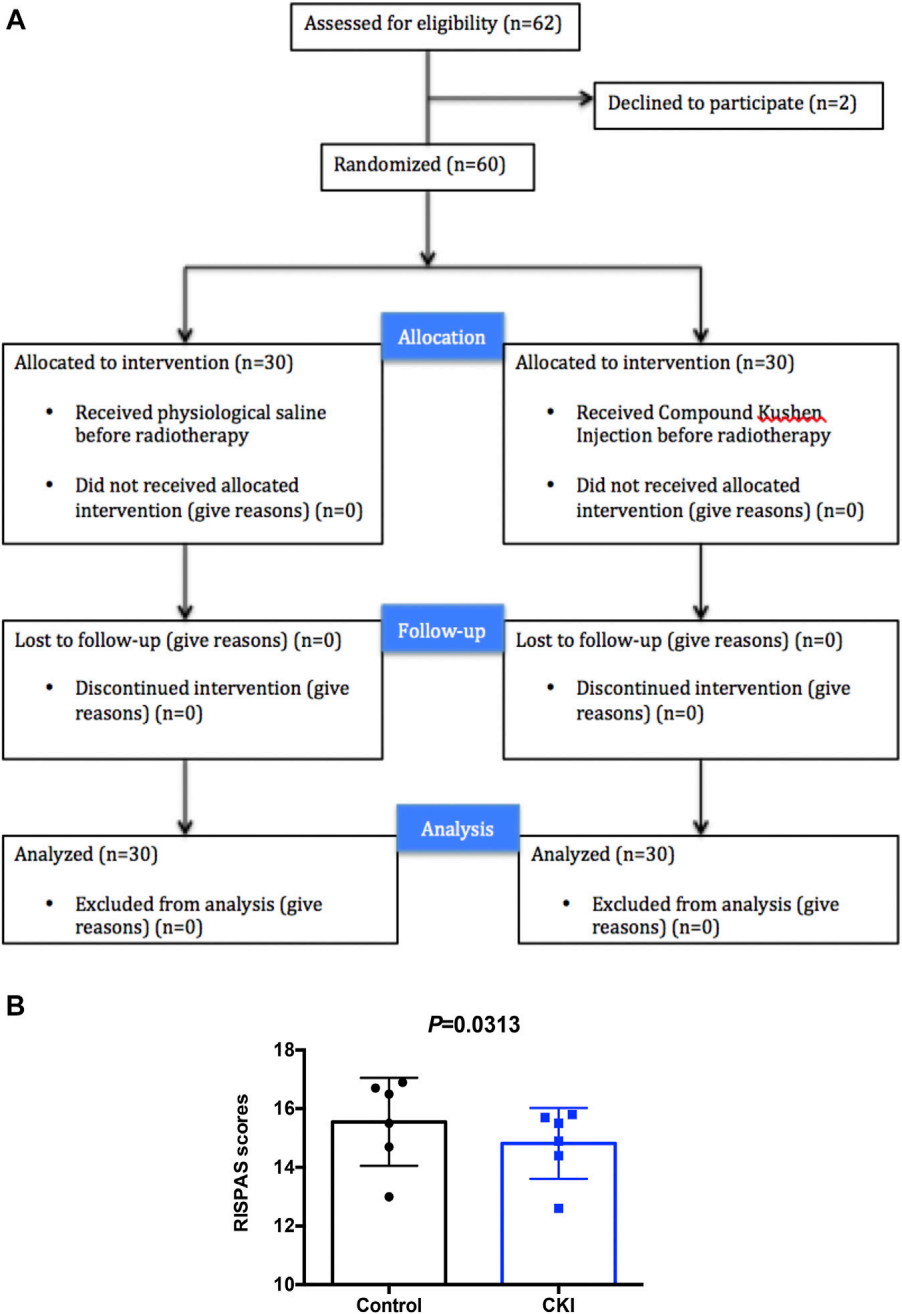
Patients had lower RISPAS scores in the CKI- treated group compared to control group. **(A)** Participant flow through the trial. **(B)** Graph showing RISPAS scores in the CKI and control group. *p* = 0.0313 (< 0.05), indicates a significant difference in the CKI group compared to the untreated control group.

A prospective randomized controlled clinical trial was conducted to investigate the difference in the severity of radiation injury of skin between the two groups of NPC patients after radiotherapy treated with or without CKI. A total of 62 NPC patients were admitted to the Department of radiotherapy of Guangdong Provincial Hospital of Chinese Medicine, from June 2014 to January 2018. In the course of treatment, 2 patients withdrew from the clinical trial due to their personal wishes. Sixty NPC patients were randomly divided into control and CKI group, both of which were accepted intensity modulated radiotherapy (IMRT). The patients from control group receivednormal saline intravenously before radiotherapy and from CKI group received CKI intravenously before radiotherapy (Figure 1A).

At the end of radiotherapy, the incidence of grade III and grade IV radiodermatitis in the CKI-treated group was 30.0 and 13.3%, respectively. While the incidence of grade III and grade IV radiodermatitis in the control group was 56.7 and 20.0%, respectively. Obviously, the data showed that the incidence of severe radiodermatitis in the CKI-treated group was lower than that in the control group, and the difference was statistically significant (*p* = 0.03, Table 1). Moreover, the RISRAS (Radiation-Induced Skin Reaction Assessent Scale) score of the CKI-treated group was also lower than that of the control group, and the difference was statistically significant (*p* = 0.002, Table 2; Figure 1B). Therefore, the above clinical results revealed that CKI could alleviate radiotherapy injury in the skin significantly in clinic.

**TABLE 1:** Radiation injury of neck skin was alleviated in CKI-treated group.

Groups	Grade-II	Grade-III	Grade-IV	χ^2^	*p* value
Control	7	17	6	7.028	0.030
CKI	17	9	5	—	

**TABLE 2:** Patients have lower RISPAS scores in the CKI-treated group compared to control group (mean ± SD).

Group	2W	3W	4W	5W	6W	7W	F	*p* value
Control	13.0 ± 0.9	14.7 ± 1.0	15.5 ± 1.2	16.5 ± 1.3	16.7 ± 1.3	16.9 ± 1.2	10.14	0.0313
CKI	12.6 ± 0.8	14.4 ± 0.9	14.9 ± 1.0	15.5 ± 1.2	15.7 ± 1.3	15.8 ± 1.2	—	

### CKI Inhibited Cancer Cell Growth While Promoted HSF Cell Growth

**FIGURE 2:**
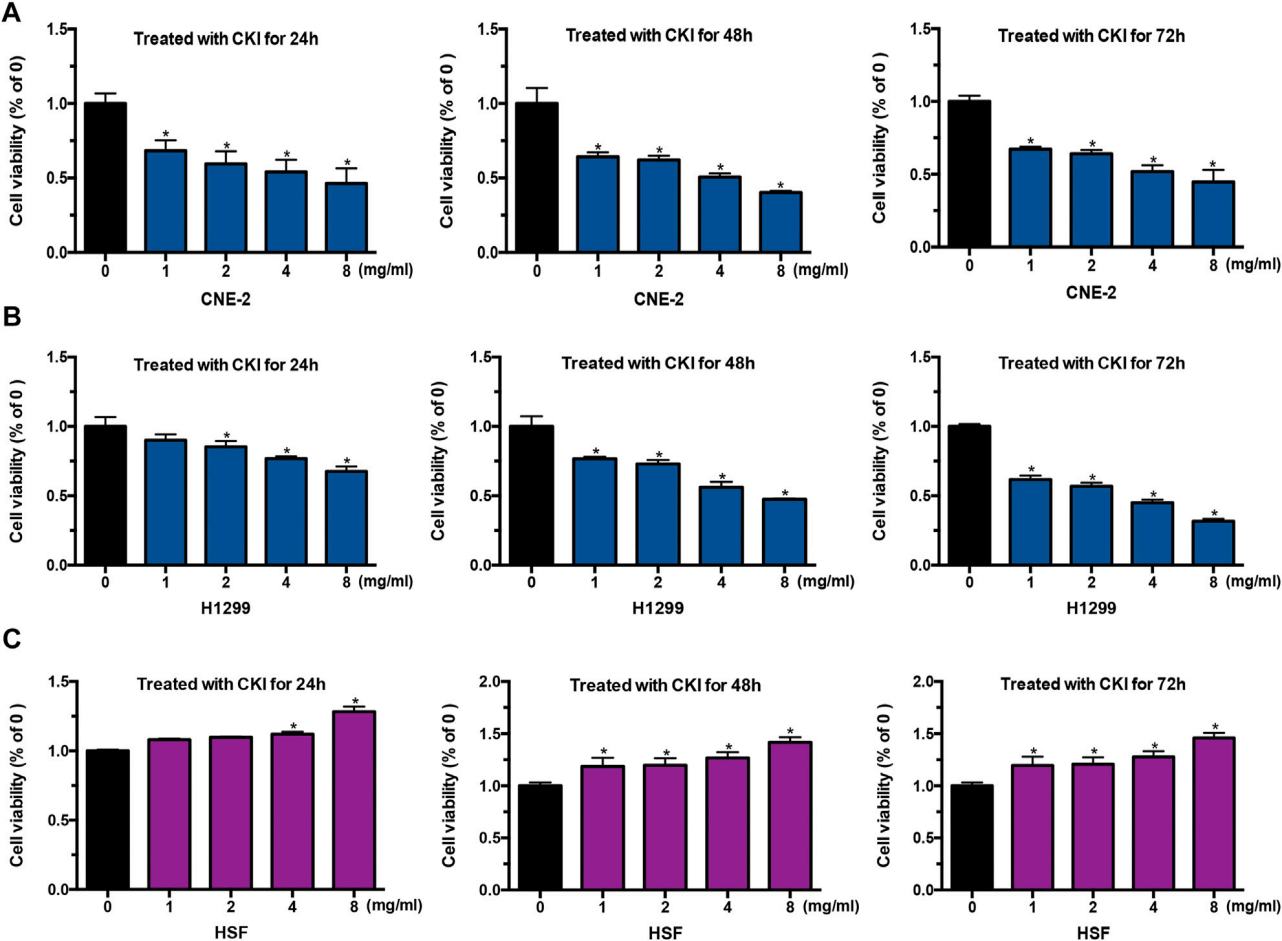
CKI inhibited tumor cell growth while promotes HSF proliferation. **(A–C)** Cancer cells (CNE-2 and H1299) and HSF cells were treated with increased concentrations of CKI for 24, 48, and 72 h. Afterwards, cells were treated with MTT as detailed in Materials and Methods. Values in bar graphs were given as the mean ± SD from three independent experiments performed in triplicate. *Indicates significant difference as compared to the untreated control group (*p < 0.05).

The above clinic data have shown that CKI had the effect of alleviating radiotherapy injury in the skin. To understand the mechanism by which CKI alleviates radiodermatitis, we first performed MTT assay to see the effect of CKI on cancer cells and HSF cells. As shown in Figures 2A,B, CKI inhibited human cancer cell growth including NPC and LC, in a both dose- and time-dependent manner. However, in the HSF cells, CKI showed a promotion effect in HSF cell growth (Figure 2C). The above results showed a differential role of CKI in human cancer and HSF cells.

### Combination of CKI and IR Inhibited Cancer Cell Growth Additively, While Protected HSF From IR Treatment

**FIGURE 3:**
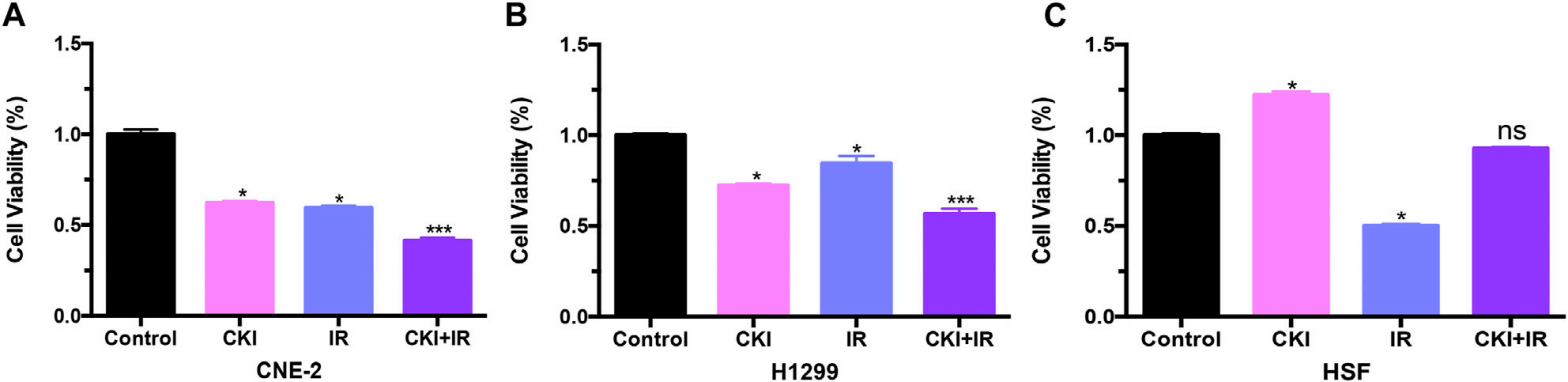
CKI combined with IR inhibited cancer cell growth additively, while had no obvious effect in HSF cells compared to control. **(A–C)** Cancer cells (CNE-2 and H1299) and HSF cells were treated with CKI (4 mg/ml), IR (4Gy), and CKI combined with IR. Afterwards, cell viability was analyzed using MTT assay. Values in bar graphs were given as the mean ± SD from three independent experiments performed in triplicate. *Indicates significant difference as compared to the untreated control group (*p < 0.05; ***, *p* < 0.001). ns, not significant, indicates no significant difference as compared to the untreated control group.

Whether CKI could influence the effect of cancer radiotherapy? We treated cancer cells and HSF cells with CKI, IR, and the combination of CKI and IR, respectively. Our findings showed an additive effect of CKI and IR in inhibiting cancer cells, as shown in Figures 3A,B. Nevertheless, there was no inhibition effect seen in the HSF cell growth under the combination treatment of CKI and IR, as shown in Figure 3C. The data showed that CKI could reverse the inhibition effect of IR in HSF cells, suggesting a protective role of CKI in HSF cells when treated with IR.

### Combination of CKI and IR Promoted Cancer Cell Apoptosis Additively, While Protected HSF From IR Treatment

**FIGURE 4:**
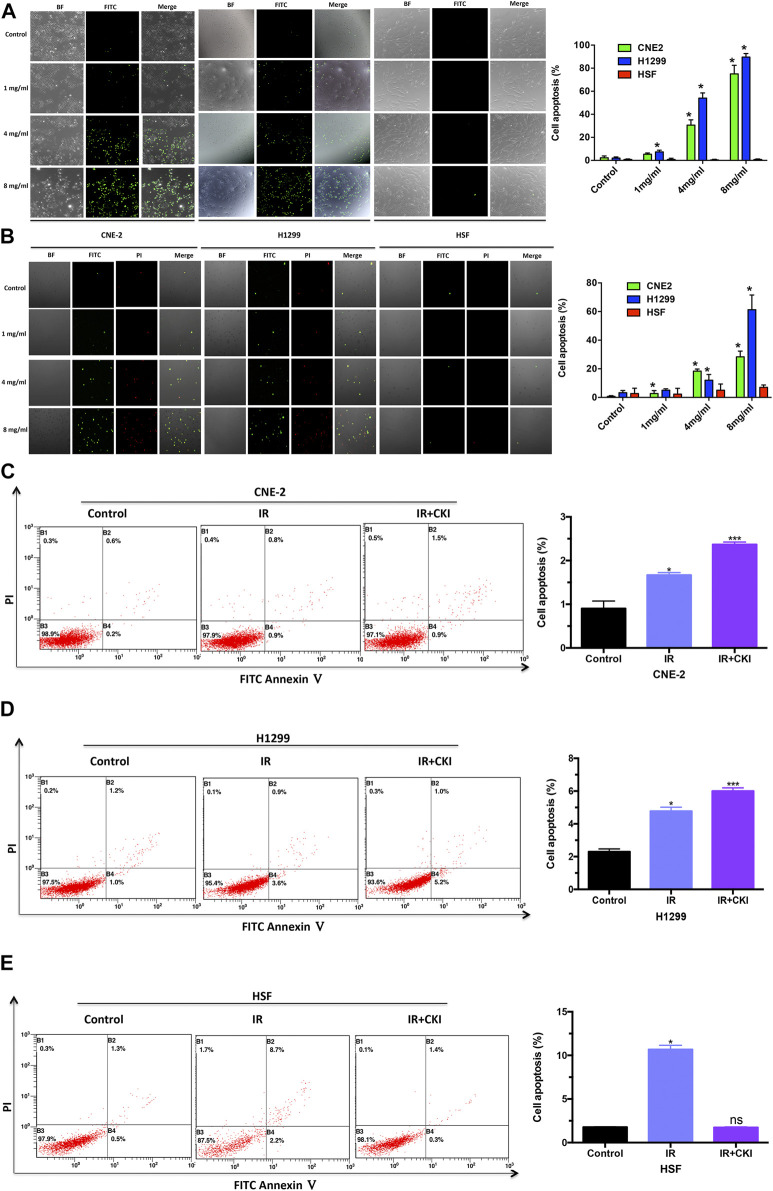
CKI enhanced cancer cell apoptosis by IR, while rescued the HSF cell apoptosis when treated with IR. **(A)** Cancer cells (CNE-2 and H1299) and HSF cells were treated with CKI (0, 1, 4, 8 mg/ml), and cell apoptosis was observed by TUNEL assay. **(B)** Cancer cells (CNE-2 and H1299) and HSF cells were treated with CKI (0, 1, 4, 8 mg/ml), and cell apoptosis was detected by doing PI and FITC annexin-V double staining using confocal microscope. **(C–E)** Cancer cells (CNE-2 and H1299) and HSF cells were treated with IR (4Gy) and CKI (4 mg/ml) combined with IR. Afterwards, cell apoptosis was analyzed using flow cytometry. Values in bar graphs were given as the mean ± SD from three independent experiments performed in triplicate. *Indicates significant difference as compared to the untreated control group (*p < 0.05; ***, *p* < 0.001). ns, not significant, indicates no significant difference as compared to the untreated control group.

TUNEL assay was performed to show the effect of CKI on cancer cells (CNE2 and H1299) and HSF cells. The results showed that CKI induced cancer cell apoptosis while has no obvious induction effect of HSF cell apoptosis (Figure 4A). Meanwhile, PI and FITC annexin-V double staining using confocal microscope was also carried to show that CKI could induce cancer cell apoptosis rather than HSF (Figure 4B). To further understand the mechanism by which CKI protects HSF cells from IR treatment. We then performed flow cytometry of cell apoptosis. As expected, CKI combined with IR had an additive effect of promoting cancer cell apoptosis (Figures 4C,D). While there was no induction effect of cell apoptosis observed in HSF cells when combined CKI with IR (Figure 4E). HSF cell apoptosis was markedly increased following IR alone. While pretreatment with CKI followed by IR resulted in reduced apoptosis levels, which had no significance compared with control group, indicating that CKI could inhibit irradiation-induced apoptosis in HSF cells. In total, the above results showed that CKI could reverse the IR-induced cell apoptosis in HSF cells, indicating a protective role of CKI in the skin.

### CKI Increased Bim Expression in Cancer Cells While had No Obvious Effect in HSF Cells

Cell apoptosis was more induced in CKI together with IR group in cancer cells, while no obvious induction of cell apoptosis was found in HSF cell, even a rescue effect of CKI was observed in HSF cells when combined with IR. Apoptosis is a highly conserved form of programmed cell death that can be triggered by extrinsic or intrinsic signals. Bcl-2 family proteins play a decisive role in apoptosis initiated by intrinsic signaling by regulating the integrity of the mitochondrial outer membrane (MOM). It is composed of three classes: pro-survival proteins (BCL-2, MCL-1, BCL-XL, BCL-w, and BFL-1), multi-domain pro-apoptotic proteins (BAX and BAK) that compromise the outer mitochondrial membrane, and BH3- only pro-apoptotic proteins (BIM and NOXA). Among those, the BH3-protein Bim (BCL-2-interacting mediators of cell death) is an important mediator of apoptosis initiated by intracellular stressors. It is related to tumor progression, metastasis, drug resistance, and promotes apoptosis at mitochondria by activating proteins Bax and Bak and by inhibiting the anti-apoptotic proteins Bcl-XL, Bcl-2, and Mcl-1 (Chi et al., 2020).

**FIGURE 5:**
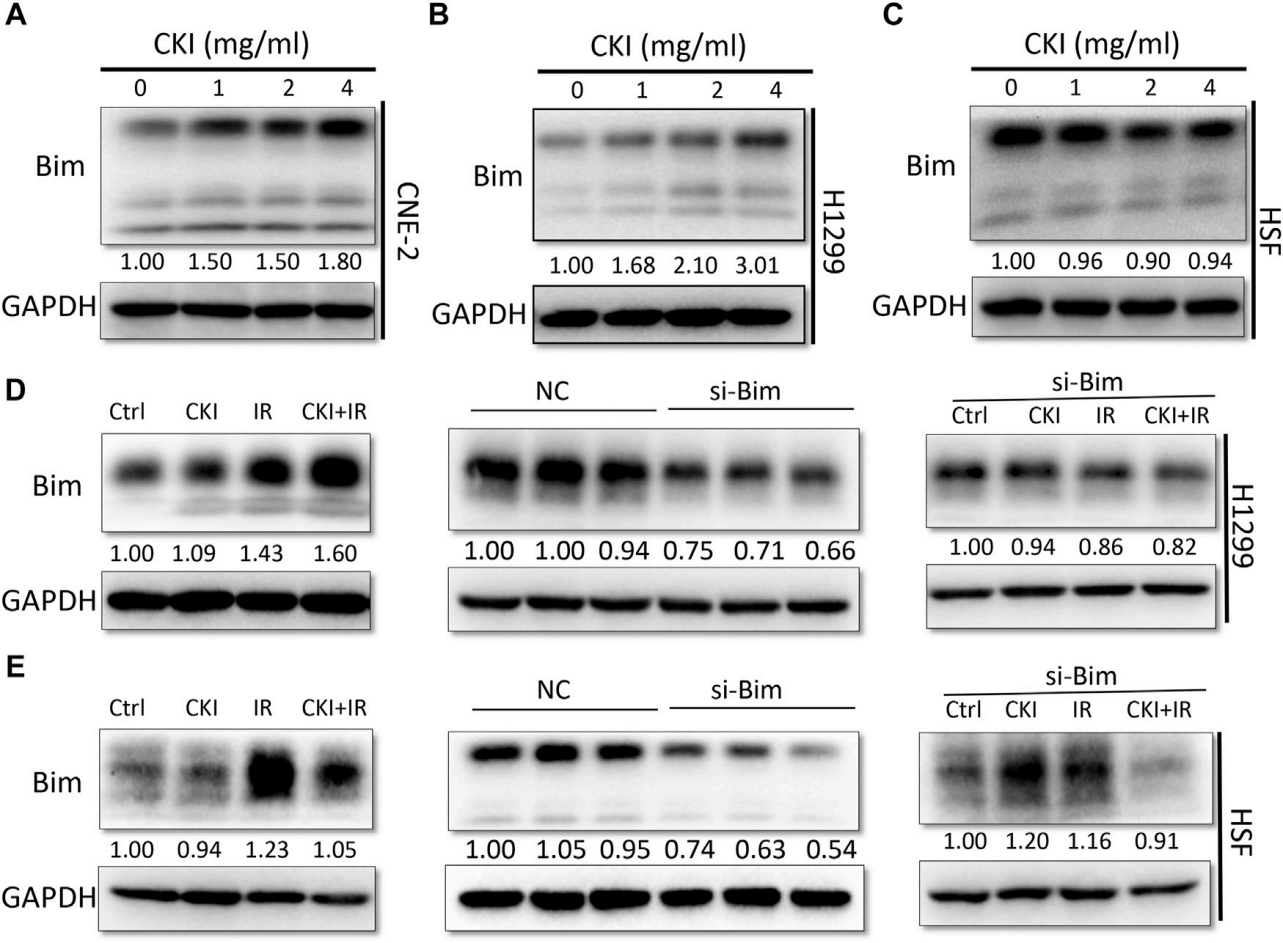
Inhibiting Bim attenuated the differential Bim expression trends caused by the combination of CKI and IR. **(A–C)** Cancer cells (CNE-2 and H1299) and HSF cells were treated with increased dose of CKI. **(D–E)** Cancer cell H1299 and HSF cells were treated with CKI (4 mg/ml), IR (4Gy) and combination of CKI and IR, respectively. Transient transfection was performed to inhibit Bim expression by siRNA. Bim expression was evaluated using Western Blot. GAPDH was used as an external control. The results were measured by ImageJ software.

Therefore, we detected Bim expression in both cancer and HSF cells when treated with CKI. Our results showed that Bim was significantly increased in both NPC and LC cells, while no significant change was observed in HSF cells, with the increased dose of CKI (Figures 5A–C). The results indicated that Bim might be a key protein leading to the differential role of CKI in cancer and HSF cells. When combined CKI with IR, a additive increase expression level of Bim was observed in H1299 cells while a slight reverse effect was found in HSF cells (Figures 5D,E, left panel). When Bim was silenced by siRNA, the differential expression trends of Bim in H1299 and HSF cells by the treatment with CKI and IR were almost disappeared (Figures 5D,E, right panel). The data suggested that Bim plays a crucial role in the differential effect of CKI in human cancer and HSF cells, especially when combined with IR.

### Silencing Bim Attenuated the Effect of CKI in Cancer and HSF Cell Apoptosis When Combined with IR

**FIGURE 6:**
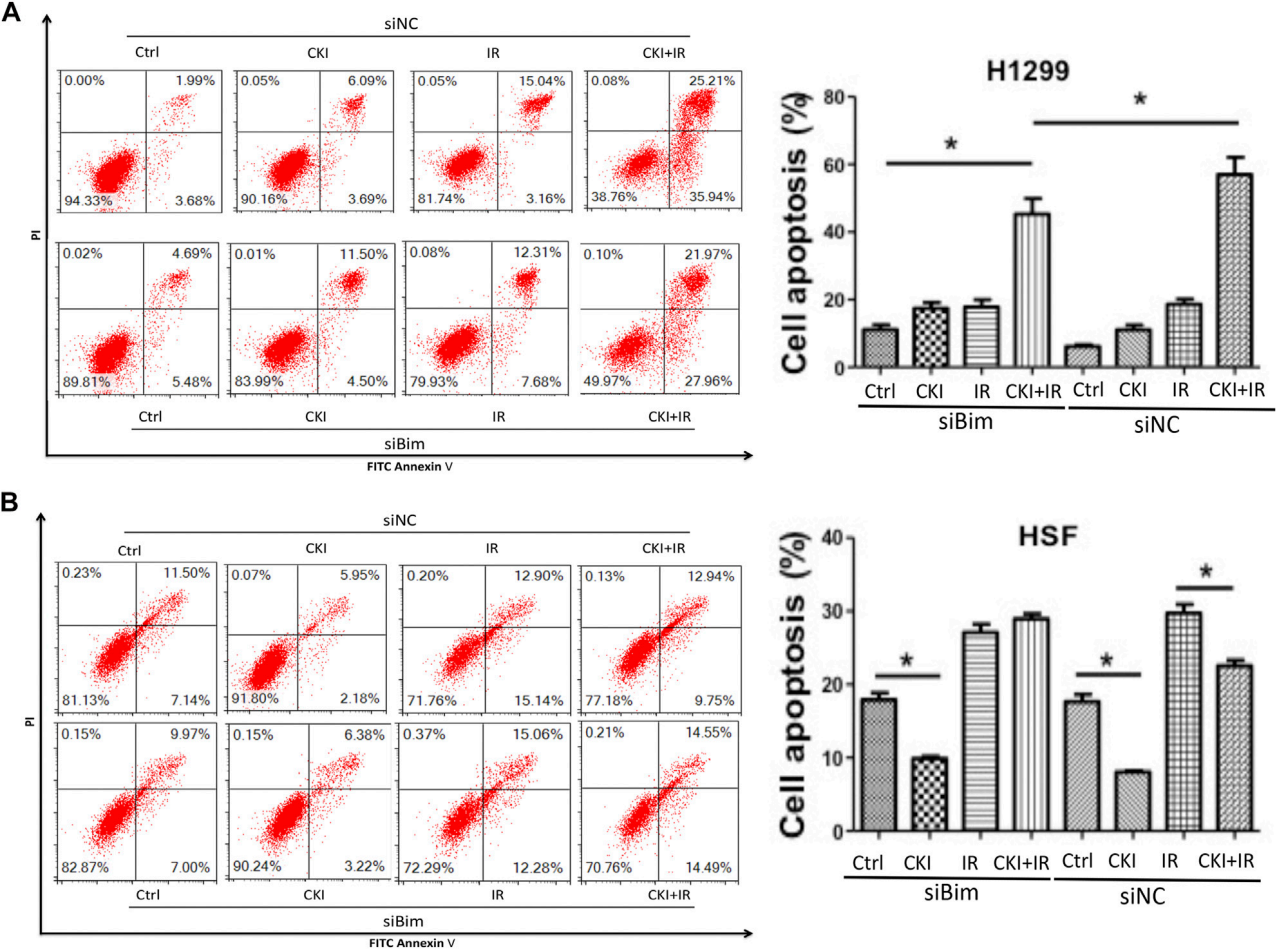
Inhibiting Bim attenuated the differential effect of CKI in cancer and HSF cell apoptosis. **(A, B)** Cancer cell H1299 and HSF cells were treated with CKI (4 mg/ml), IR (4Gy) and combination of CKI and IR, respectively. Cell apoptosis was analyzed using flow cytometry. Values in bar graphs were given as the mean ± SD from three independent experiments performed in triplicate. *Indicates significant difference as compared to the accordingly group (*p < 0.05).

Since the differential expression of Bim almost disappeared when inhibiting Bim in both cancer cells H1299 and HSF cells, whether the cell apoptosis effect could be suppressed when Bim was silenced is to be identified. We then performed flow cytometry to detect the cell apoptosis percentages by inhibiting Bim expression. Our data showed that in H1299 cells, inhibiting Bim could partially reverse the combination effect of CKI and IR in inducing cell apoptosis (Figure 6A). And in HSF cells, inhibiting Bim attenuated the effect of CKI combined with IR in reversing the cell apoptosis by IR (Figure 6B). This data reconfirmed that Bim is the key protein making CKI functions differently in cancer and HSF cells. Therefore, we concluded that CKI protected skin from radiation injury *via* regulating Bim.

## Discussion

Many studies have shown that CKI could relieve acute radiation injury and protect normal tissue of patients. The protective effects of CKI on human dermal fibroblasts suggest that it has potential applications in the protection against irradiation -induced skin injury. However, the mechanism of CKI in reducing radiation injury is unknown. In the present study, we are proposed to uncover the mechanisms by which CKI protect skin against radiation injury.

In the present study, we first identified that CKI had the effect of alleviating radiotherapy injury in the skin in patients with NPC who received IMRT. To discover the mechanism, we then performed a series of *in vitro* experiments. We found that CKI inhibited cancer cell growth by inducing cell apoptosis in NPC CNE-2 cells and LC H1299 cells. At the same time, CKI promoted HSF cell proliferation in a dose- and time-dependent manner. Apoptosis plays a key role in the differential role of CKI in cancer and non-cancerous cells. The induction of apoptosis is a common and required event for different classes of anticancer agents, and disruption of such mechanism can lead to non-specific side effects (Shukla et al., 2017). Our further data found that Bim is the key protein making CKI plays a differential role of in human cancer and HSF cells when combined with IR.

Bim, also known as B-cell chronic lymphocytic leukemia/lymphoma (Bcl-2)-like 11 (BCL2L11), is a member of the Bcl-2 family and a critical modulator of cell apoptosis. Bim encodes the BH3 protein, which activates cell death either by opposing the pro-survival activities of members of the BCL2 family or by binding to and directly activating pro-apoptotic BCL2 family members. It promotes apoptosis by activating Bax/Bak, and it is regulated at both transcriptional and post-translational levels (Byerly et al., 2020). Induction of Bim triggers cytochrome C release from mitochondria to cytosol. Then, cytosolic cytochrome C induces Caspase cleavage followed by PARP cleavage, finally resulting in cell apoptosis (Akiyama et al., 2009). The overexpression of Bim inhibits tumor growth and drug resistance. Cancer cells suppress Bim expression, associating with tumor promotion, metastasis, and drug resistance (Akiyama et al., 2009; Shukla et al., 2017). For example, Bim plays an important role in sensitizing epidermal growth factor receptor-tyrosine kinase inhibitors (EGFR-TKIs) in EGFR-Positive NSCLC (Incharoen et al., 2019). And modulating Bim transcription is one of the mechanisms by which aspirin overcomes osimertinib resistance in EGFR-mutated NSCLC (Han et al., 2020). A study reported that the homozygous deletion of Bim characterizes it as a novel tumor suppressor in MCL (mantle cell lymphoma) (Tagawa et al., 2005). MCL with high levels of proapoptotic Bim expression are more likely to result in a patient’s complete response rather than progressive disease following therapy (Wang JD. et al., 2019). In accordance with other hematopoietic and solid-organ malignancies, Bim’s role as a tumor suppressor appears to have prognostic and therapeutic significance. For example, in chronic myeloid leukemia (CML), Bim deletion polymorphisms lengthen the time for patients to achieve a major molecular response on TKIs (Than et al., 2019). Additionally, loss of Bim or its expression in melanoma, colorectal cancer, intrahepatic cholangiocarcinoma, and EGFR-positive NSCLC is associated with worse prognoses (Dai et al., 2008; Sinicrope et al., 2008; Ng et al., 2012; Lee et al., 2015; Zhang et al., 2018). Those data revealed the important role of Bim in human cancers and cancer-related therapies. Here in the present study, Bim serves as a critical protein in protecting skin from radiation injury, with the advent of radiotherapy acting on the cell apoptosis.

## Conclusion

Our study indicated that CKI represents a promising radioprotective agent with a potential differential beneficial effect on both cancer cells (inducing apoptosis) and HSF cells (providing radio-protection *via* inhibiting IR-induced apoptosis) as clearly demonstrated through this study, *via* regulation of mitochondria pathway by regulating Bim. Taken together, these results uncover a novel mechanism by which CKI inhibits human cancer while protect skin from radiotherapy.

## Data Availability

The raw data supporting the conclusion of this article will be made available by the authors, without undue reservation.
