# Supplementary material for: Compound Kushen Injection Protects Skin From Radiation Injury via Regulating Bim
Source: Front Pharmacol. 2021 Dec 9;12:753068. doi: 10.3389/fphar.2021.753068 (PMC8696473; doi:10.3389/fphar.2021.753068)
Supplement: Supplementary file 1 [file Table1.DOCX]

Supplementary tables

Table 1. RISRAS of subjective symptom perception scale by patients

Symtoms not at all a little some very

Do you feel any tension,

discomfort or pain 0 1 2 3

in the radiation field skin?

Do you feel itchy? 0 1 2 3

Do you feel burning on the 0 1 2 3

skin of the radiation site?

To what extent do you feel

that skin reactions or symptoms 0 1 2 3

caused by radiotherapy

affect your daily activities?

RISRAS: The Radiation-Induced Skin Reaction Assessent Scale

Table 2. RISRAS of objective symptom score by medical staff

Symtoms 0 1 2 3 4

Erythema (E) Normal Pallidum E Gray E Bright E Deep purple E

Dry desquamation Normal <25% <25%-50% >50%-75% >75%-100%

Moist desquamation Normal <25% <25%-50% >50%-75% >75%-100%

Necrosis Normal <25% <25%-50% >50%-75% >75%-100%

RISRAS: The Radiation-Induced Skin Reaction Assessent Scale
